# Large-scale experimental investigation of biotreated sand column using different grouting pipe configurations

**DOI:** 10.1371/journal.pone.0349797

**Published:** 2026-05-26

**Authors:** Chunyan Wang, Zhikang Wei, Jinquan Shi, Xiang He

**Affiliations:** 1 School of Civil Engineering, Chongqing University, Chongqing, China; 2 Department of Civil and Environmental Engineering, Hong Kong University of Science and Technology, Hong Kong, China; NED University of Engineering and Technology, PAKISTAN

## Abstract

Microbially induced carbonate precipitation (MICP) has considerable potential for applications such as soil improvement, erosion control, and heavy metal remediation. However, the extent to which grouting pipe configuration affects treatment performance remains unclear. To address this gap, this study investigated the effects of grouting pipe configuration on the reinforcement behaviour of large-scale biotreated sand columns. Large-scale model tests were conducted using conventional vertical pipes and a newly proposed spiral pipe under different grouting procedures. Calcium carbonate content, unconfined compressive strength, penetration resistance, and microscopic characteristics were analysed to evaluate reinforcement range, spatial uniformity, and treatment depth. The results show that pipe configuration plays a key role in controlling calcium carbonate distribution and reinforcement performance. The one-pipe-injection-extraction configuration produced a limited effective reinforcement radius of about 0.09 m, whereas both the three-injection-one-extraction and spiral-injection-one-extraction configurations increased this value to about 0.15 m. Compared with conventional vertical pipes, the spiral configuration produced a more uniform distribution of calcium carbonate content and strength by reducing local enrichment and weakly treated zones. For the 1.5 m-high specimen, relatively uniform reinforcement was mainly achieved within the upper 0.6 m under the present pressure level. This study provides a practical pipe-layout strategy for improving the uniformity of field-scale MICP treatment and offers useful guidance for the design of bio-grouting systems.

## Introduction

Microbial geotechnics is an emerging interdisciplinary field that exploits microbial activity to modify the mechanical and hydraulic behaviour of soils, including strength, stiffness, and permeability [1–5]. It has been widely recognised as a promising research direction in geotechnical engineering in the twenty-first century [6,7]. Among the various bio-mediated techniques, Microbially Induced Carbonate Precipitation (MICP) has attracted particular attention owing to its high cementation efficiency and low energy demand [3,8]. Extensive studies have been conducted on the cementation mechanisms, mechanical behaviour, and influencing factors associated with MICP-treated soils [4,7,9–11]. However, most existing investigations rely on small laboratory specimens, typically with diameters smaller than 100 mm and heights less than 250 mm. Representative examples include microfluidic tests [12–14], particle-scale observations [15–17], and element-scale experiments [18–20]. These studies have significantly advanced the understanding of MICP mechanisms and established a solid theoretical foundation for the technique. Nevertheless, small-scale tests generally employ one-dimensional injection schemes, and the associated solution transport paths and cementation patterns differ fundamentally from the three-dimensional grouting conditions encountered in engineering practice [21,22]. As a result, their direct applicability to field grouting design, particularly in relation to pipe arrangement and operational parameters, remains limited.

Large-scale experiments offer improved representation of the spatial geometry and boundary conditions governing solution transport and reaction processes during MICP treatment, and therefore provide more relevant insights for field application [23–25]. Whiffin et al. [26] conducted MICP treatment on a sand column 5 m in length and 66 mm in diameter, demonstrating that reductions in porosity and increases in calcium carbonate content and strength decreased progressively with distance from the injection point. van Paassen et al. [27] subsequently applied MICP to a 100 m^3^ sand foundation model, with injection and extraction wells installed at opposite ends of the test box and bacterial and cementation solutions injected alternately. After ten days of treatment, effective cementation was confined to approximately 40 m^3^ of sand located between the wells, indicating a strong dependence of reinforcement extent and spatial uniformity on the layout and spacing of injection and extraction wells. Sharma et al. [28] investigated a sand model measuring 1.35 m × 1.13 m × 0.65 m and showed that appropriate optimisation of injection spacing mitigated localised accumulation of calcium carbonate. Gomez et al. [29] and Nassar et al. [30] treated sand foundations with diameters of 1.7 m and heights of 0.3 m, reporting that calcium carbonate preferentially accumulated along dominant seepage pathways, while bacterial and cementation solution concentrations decreased with increasing flow distance. Sang et al. [24] examined a sand specimen 1 m in diameter and 0.15 m in height, and demonstrated, through unconfined compressive strength testing and calcium carbonate measurements, that soil heterogeneity and preferential flow paths exerted a strong influence on the spatial distribution of strength and cementation.

Although these large-scale studies have improved understanding of the mechanisms responsible for non-uniformity during MICP treatment, most were conducted using a single grouting strategy and a fixed pipe layout [31,32]. Systematic investigations remain limited regarding how grouting pipe configuration and treatment procedure jointly affect calcium carbonate distribution, strength development, and the effective cementation range in large-scale specimens [33]. Because pipe configuration and treatment procedure are two of the key design variables in practical grouting applications, understanding their effects is important for the design and optimizing biogrouting performance.

To address these gaps, this study presents a series of large-scale MICP grouting experiments on sand columns with a diameter of 0.3 m and heights of 0.4 m and 1.5 m. Conventional vertical pipes and a newly proposed spiral pipe configuration were comparatively evaluated using one-phase and two-phase treatment methods. Particular attention was paid to treatment performance under constant-pressure grouting and synchronized injection-extraction conditions. The specific objectives were to: (1) compare the effects of different grouting pipe configurations and treatment procedures on the spatial distributions of calcium carbonate content and strength; (2) define and quantify the effective reinforcement radius based on a measurable cementation criterion; and (3) evaluate the feasibility of forming large-scale MICP-treated sand columns using spiral-injection-one-extraction pipes. The findings are expected to provide both mechanistic insight and practical guidance for the design of field-scale MICP grouting systems.

## Materials and methods

### Testing materials

**Fig 1:**
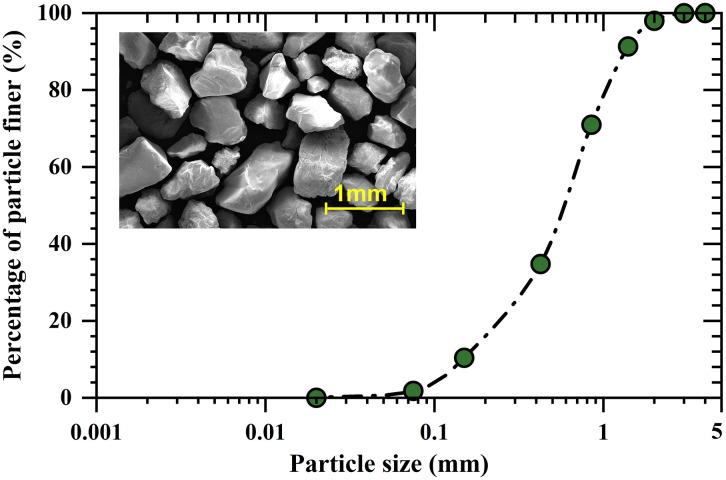
Particle size distribution of testing sand.

The sand used in the experiments was Fujian standard sand, with a mean particle size *d*_*50*_ of 0.6 mm. The coefficient of uniformity and coefficient of curvature were 4.0 and 1.78, respectively. The particle size distribution curve and representative microstructural features are shown in **Fig 1**, and the basic physical and mechanical properties of the sand are summarised in **Table 1**. *Sporosarcina pasteurii* (ATCC 11859) was selected as the urease-producing bacterium and was activated and cultured to obtain bacterial suspensions with urease activities ranging from 12 to 16 U. Industrial-grade urea and calcium chloride were used to prepare the cementation solution, with equimolar concentrations of urea and calcium chloride maintained at 1 mol/L [27].

**Table 1:** Physical properties of silica sand.

Physical properties	Values
Maximum dry density, $\rho_{dmax}$ ($g/cm^{\text{3}}$)	1.64
Minimum dry density, $\rho_{dmin}$ ($g/cm^{\text{3}}$)	1.35
Maximum void ratio, $e_{max}$	0.95
Minimum void ratio, $e_{min}$	0.60
Specific gravity, $G_{s}$	2.63
Frictional angle, φ: °	32

### Biogrout preparation and biogrouting pile fabrication

**Fig 2:**
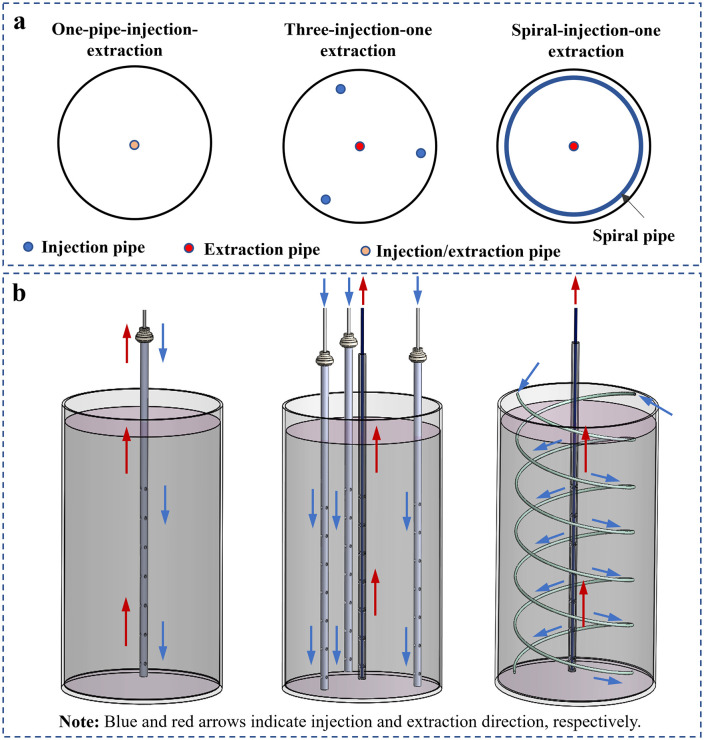
Layout of grouting pipes: (a) 2D diagram; (b) 3D schematic.

Five large-scale MICP-treated sand column model tests were conducted, incorporating three grouting pipe configurations and two treatment procedures. The experimental programme is summarised in **Table 2**. All sand columns had a diameter of 0.3 m, with heights of 0.4 m and 1.5 m. The grouting pipe configurations, as illustrated in **Fig 2**, comprised the one-pipe-injection-extraction configuration, the three-injection–one-extraction configuration, and the spiral-injection-one-extraction configuration. In the one-pipe-injection-extraction configuration, a single vertical pipe located at the centre of the sand column was used for both injection and extraction. The three-injection–one-extraction configuration consisted of four vertical pipes, with a central extraction pipe surrounded by three uniformly spaced injection pipes. In the spiral-injection-one-extraction configuration, a central vertical extraction pipe was installed, together with two spiral injection pipes arranged around it. The vertical injection and extraction pipes were fabricated from PVC with an inner diameter of 17 mm and an outer diameter of 20 mm [34]. Injection pipes were perforated with 2 mm outlets arranged in three circumferential directions at a vertical spacing of 2 cm over a perforated length of 0.35 m. Extraction pipes were perforated symmetrically in four directions, with inlet diameters of 5 mm at 3 cm spacing over a perforated length of 0.30 m. The spiral injection pipes were made of transparent polyurethane hoses with an inner diameter of 5 mm and an outer diameter of 8 mm, with uniformly distributed 2 mm outlets along a perforated length of 0.35 m. To eliminate clogging during grouting, all pipes were wrapped with a layer of nonwoven scouring fabric [35]. In **Fig 2**, blue and red arrows indicate injection and extraction direction respectively, and the helical path of the spiral injection pipe is explicitly illustrated.

**Table 2:** Experimental schemes.

Test ID	Grout pipe configurations	Treatment method	Dimensions of biotreated sand column	Measured parameters	Treatment cycles
U1	One-pipe-injection-extraction	Low-pH one-phase	D = 0.3m, L = 0.4m	Calcium carbonate content	10
U2	One-pipe-injection-extraction	Two-phase	D = 0.3m, L = 0.4m	Calcium carbonate content	10
U3	Three-injection-one-extraction	Two-phase	D = 0.3m, L = 0.4m	Calcium carbonate content, Unconfined compressive strength	10
U4	Spiral-injection-one-extraction	Two-phase	D = 0.3m, L = 0.4m		10
U5	Spiral-injection-one extraction	Two-phase	D = 0.3m, L = 1.5m	Calcium carbonate content, penetration strength	15

A constant-pressure injection mode was employed to deliver the biogrout so as to fabricate the sand column. A grouting pump with a fixed flow rate of 8 L/h was used to sustain the injection pressure at approximately 0.02 MPa throughout the test. Injection and extraction were conducted synchronously under controlled conditions, and this operational mode was adopted in the present tests to promote solution replacement and maintain relatively stable treatment conditions. The selected pressure level was intended to provide sufficient solution transport while avoiding excessive disturbance to the sand column.

**Fig 3:**
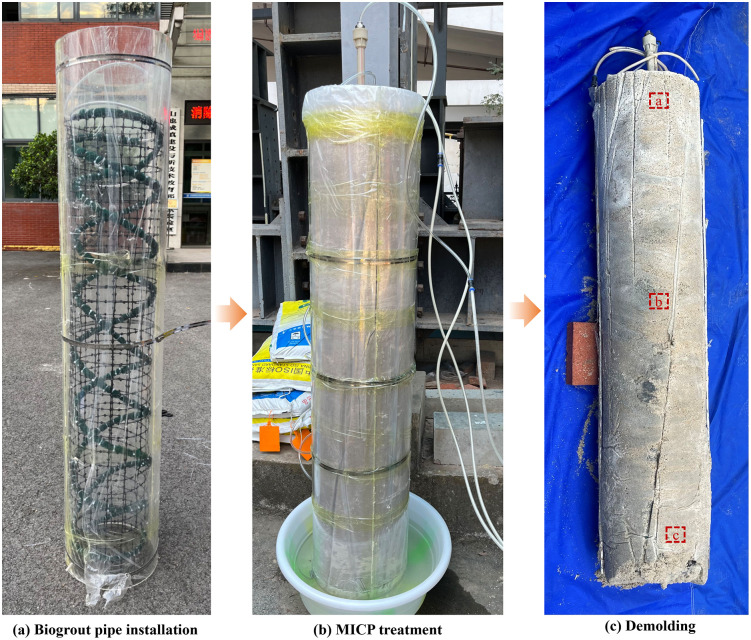
Fabrication of 1.5 m spiral-grouted sand column.

The influence of treatment procedure was investigated by comparing specimens U1 and U2, which employed the same one-pipe-injection-extraction configuration. Specimen U1 was treated using the low-pH one-phase method [36], whereas specimen U2 was treated using the two-phase method [37]. The effect of grouting pipe configuration was examined by comparing specimens U2, U3, and U4, all treated using the two-phase method but adopting the one-pipe-injection-extraction, three-injection-one-extraction, and spiral-injection-one-extraction configurations, respectively. In addition, specimen U5, with a diameter of 0.3 m and a height of 1.5 m, was prepared using the spiral-injection-one-extraction configuration to assess its applicability to full-scale sand column formation. Owing to its greater height, the spiral injection pipes in specimen U5 were fixed using a geogrid mesh, as shown in **Fig 3**. All sand columns were prepared by layered compaction, with the relative density controlled at 75%.

For the low-pH one-phase method, the bacterial suspension was first adjusted to a pH of approximately 5 using 1 mol/L HCl [36]. The bacterial suspension and cementation solution were then mixed at a volume ratio of 1:1 and injected simultaneously into the sand column, with a total injection volume of approximately 1.3 times the pore volume. After injection, the specimen was left undisturbed for 24 h to allow the reaction to proceed, after which the residual solution was extracted and replaced with fresh mixed solution [34]. This sequence constituted one treatment cycle. For the two-phase method, the bacterial suspension was first injected and left for 4 h to promote bacterial attachment to the sand particle surfaces [31,36]. The bacterial solution was then extracted through the central extraction pipe while the cementation solution was injected to replace it, followed by a resting period of 20 h. This procedure also constituted one treatment cycle. A total of 10 treatment cycles were applied to the 0.4 m-high sand columns, whereas 15 cycles were applied to the 1.5 m-high sand column.

### Strength and calcium carbonate content

After completion of treatment, calcium carbonate contents of sand columns U1 and U2 were determined using an acid dissolution method [26] with hydrochloric acid at a concentration of 1 mol/L. Prior to measurement, the surface calcium carbonate layer formed at the top of each sand column was removed. Sampling locations were selected on the vertical symmetry plane of each column, with horizontal and vertical spacings of 0.03 m and 0.05 m, respectively.

The strength of the treated material was evaluated using sand columns U3 and U4. These columns were sectioned into rectangular blocks measuring 50 × 50 × 100 mm, and the original spatial positions of all specimens were recorded. Unconfined compressive strength tests were then performed to characterise the spatial distribution of strength within the treated sand. Each sand column was divided into four layers of equal height, with each layer further subdivided into eight specimens of identical dimensions. The axial loading rate in the unconfined compression tests was maintained at 2 mm/min. Following mechanical testing, calcium carbonate contents of specimens from U3 and U4 were also measured using the acid dissolution method. Sampling from the rectangular blocks followed the same spatial arrangement adopted for U1 and U2, with horizontal and vertical spacings of 0.03 m and 0.05 m, respectively. To reduce the influence of occasional outliers associated with specimen cutting and local heterogeneity, the maximum and minimum values among the eight specimens in each layer were excluded, and the remaining six measurements were retained for subsequent analysis.

Sand column U5 was prepared with the spiral-injection-one-extraction pipes fixed using a geogrid mesh. Deformation induced during treatment prevented the column from being cut into standard test specimens (**Fig 3b**). Surface penetration tests were therefore adopted to evaluate the spatial variation in strength. A mortar penetration apparatus (SJY-800B, Beijing Haichuang Gaoke; resolution 0.01 mm, measurement range 20 mm) was employed. During testing, specimen U5 was placed horizontally on the ground (**Fig 3c**). Two half-column regions were randomly selected along the diameter, and each region was divided into 14 layers of equal height along the depth direction, with measurement points marked accordingly. The penetration needle was driven into each marked location, and the resulting penetration depth was recorded as an index of local strength.

In this study, the effective reinforcement radius was defined as the radial extent within which the treated sand particles were sufficiently cemented to form an integral body without loosening or collapse after demoulding. Based on the calcium carbonate measurements and the corresponding post-demoulding morphology, a CaCO_3_ content of 3% was adopted as the threshold for effective cementation [26]. Accordingly, the effective reinforcement radius was determined by the boundary corresponding to CaCO_3_ ≥ 3% [38].

## Experimental results

### One-pipe-injection-extraction configuration

**Fig 4:**
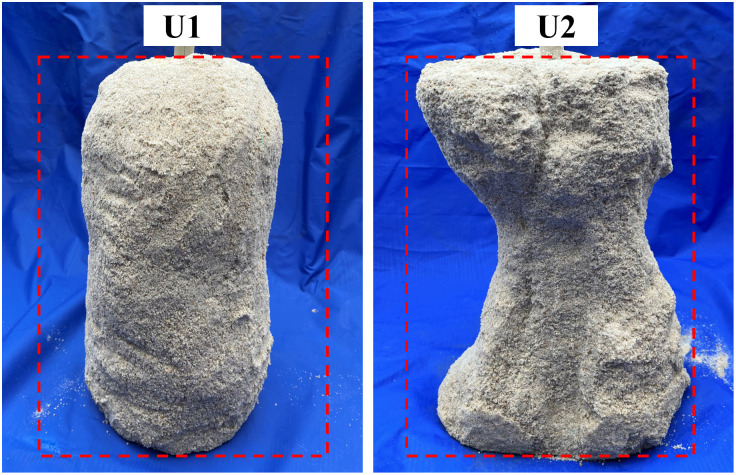
Sand columns treated using a single-tube injection–extraction method.

**Fig 4** presents the sand columns treated using the one-pipe-injection-extraction configuration (specimens U1 and U2). The red dashed outline indicates the original dimensions of the sand column, while the enclosed region shows the actual shape of the cemented body after demoulding. For both specimens, the dimensions of the cemented body were markedly smaller than the original mould size, indicating that this grouting configuration did not achieve effective cementation over the entire soil volume. Specimen U1, treated using the low-pH one-phase method, retained an approximately cylindrical shape after demoulding, with a diameter of about 0.18 m (**Fig 4**). The geometry closely resembles that reported by Martin et al. [39] for EICP-treated specimens produced using a central injection scheme. By contrast, specimen U2, treated using the two-phase method, exhibited a pronounced hourglass-shaped profile, with diameters of approximately 0.25 m at the top and bottom and about 0.15 m at mid-height.

**Fig 5:**
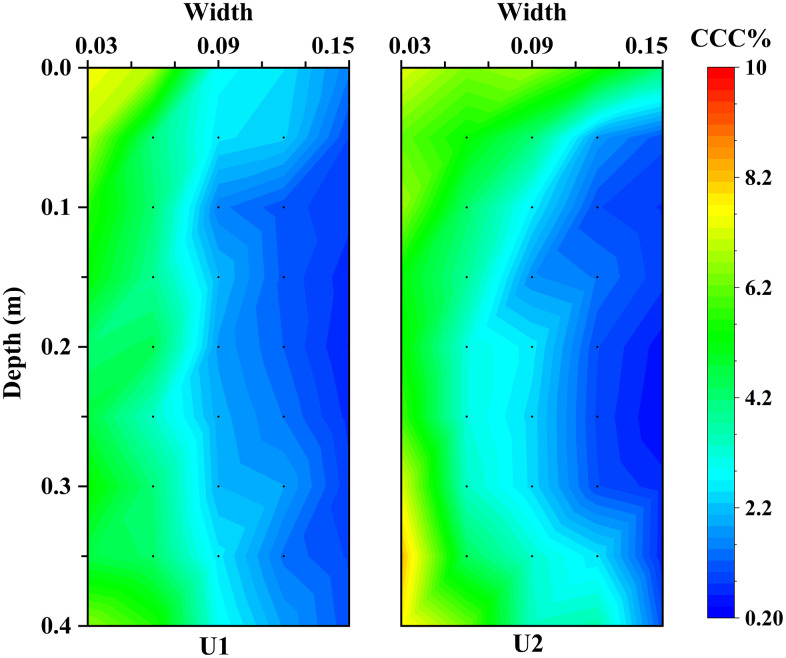
Distribution of calcium carbonate in U1 and U2.

**Fig 5** shows the spatial distribution of calcium carbonate content in specimens U1 and U2 along both the radial and depth directions. In specimen U1, calcium carbonate was predominantly concentrated within a radius of approximately 0.09 m from the grouting pipe, with a clear decreasing trend toward the column perimeter. The corresponding data are provided in S1 Table. High contents were consistently observed near the pipe, whereas values at the outer boundary were markedly lower. According to the definition adopted in this study, the effective reinforcement radius of specimen U1 was therefore approximately 0.09 m. Specimen U2 exhibited a similar radial decay pattern; however, a distinct concave zone was evident in the central region, where calcium carbonate contents were uniformly below 3%. Comparison of the cemented morphology in **Fig 4** with the chemical distribution in **Fig 5** indicates that calcium carbonate contents below approximately 3% were insufficient to generate effective interparticle bonding and a structurally continuous cemented body. The effective reinforcement range identified from the demoulded morphology is therefore consistent with the CaCO_3_-based criterion adopted in this study.

### Three-injection–one-extraction and spiral-injection-one-extraction configurations

**Fig 6:**
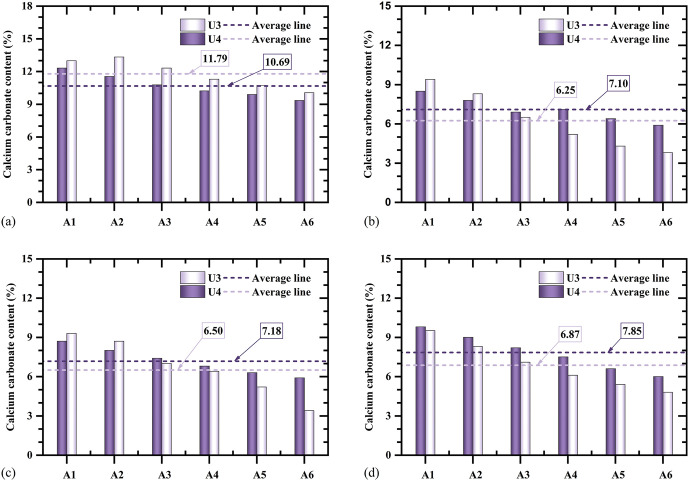
Comparisons of calcium carbonate content between U3 and U4: (a) first layer, (b) second layer, (c) third layer, and (d) fourth layer.

Sand columns treated using the three-injection–one-extraction configuration (U3) and the spiral-injection-one-extraction configuration (U4) both formed well-defined cylindrical cemented bodies after ten treatment cycles, with no visible loose zones following demoulding. Based on the CaCO_3_ ≥ 3% criterion, the effective reinforcement radius of both configurations was approximately 0.15 m. **Fig 6** illustrates the depth-wise distribution of calcium carbonate content for specimens U3 and U4 and the corresponding data are provided in S2 Table. Both specimens exhibit similar overall trends, characterised by the highest calcium carbonate contents in the top layer, significantly lower values in the middle layers, and slightly increased contents near the base.

For specimen U3, the mean calcium carbonate contents of the four layers were 11.79% (Layer 1), 6.25% (Layer 2), 6.50% (Layer 3), and 6.87% (Layer 4). The top layer content was approximately 1.87, 1.81, and 1.72 times those of Layers 2–4, respectively. The pronounced enrichment at the surface is attributed to the accumulation of excess injected solution exceeding the pore volume (≥0.3 pore volumes), which promoted preferential precipitation and retention of calcium carbonate in the upper zone [40]. From Layer 2 to Layer 4, the gradual increase in calcium carbonate content with depth is likely associated with downward migration of suspended or weakly attached carbonate precipitates driven by injection pressure and gravity, followed by accumulation near the extraction outlet [28].

Comparison between specimens U3 and U4 indicates systematic differences associated with pipe configuration. Apart from the surface layer, the average calcium carbonate contents in Layers 2–4 of specimen U4 were consistently higher than those in specimen U3. As shown in **Fig 6**, the surface-layer calcium carbonate content of U3 (11.79%) exceeded that of U4 (10.69%). In contrast, calcium carbonate contents in U4 exceeded those of U3 by 13.6%, 10.46%, and 14.26% in Layers 2–4, respectively. This redistribution pattern suggests that the spiral-injection-one-extraction configuration suppresses excessive surface accumulation while promoting more effective cementation in the middle and lower zones. Although both configurations achieved a similar effective reinforcement radius, the spiral arrangement resulted in a more uniform spatial distribution of cementation, indicating that pipe geometry influences not only the treatment extent but also the internal homogeneity of the cemented body.

Spatial variability within each layer was further quantified using the standard deviation of calcium carbonate content. Specimen U3 exhibited an overall standard deviation of 2.86%, with layer-wise values of 1.30, 2.24, 2.19, and 1.79%. In contrast, specimen U4 showed a markedly lower overall standard deviation of 1.80%, with corresponding layer-wise values of 1.10, 0.94, 1.06, and 1.44%. These results demonstrate the superior uniformity achieved using the spiral-injection-one-extraction configuration.

**Fig 7:**
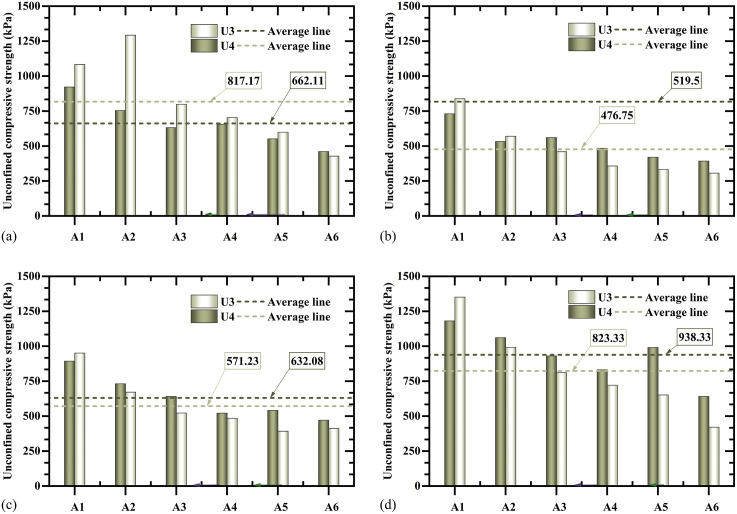
Comparisons of unconfined compressive strength between U3 and U4: (a) first layer, (b) second layer, (c) third layer, and (d) fourth layer.

**Fig 7** presents the unconfined compressive strength results for specimens U3 and U4. For both configurations, the highest average strengths occurred in the bottom layer, followed by the surface layer, while the middle layers exhibited relatively lower strengths. The corresponding data are provided in S3 Table.In specimen U4, the mean strengths of Layers 1–4 were 662.11, 519.50, 632.08, and 938.33 kPa, respectively. Relative to the surface layer, the strength ratios of Layers 2–4 were 0.78, 0.95, and 1.42.

Direct comparison indicates that specimen U3 exhibited a higher average surface strength (817.17 kPa) than specimen U4 (662.11 kPa). However, in Layers 2–4, specimen U4 consistently outperformed U3, with average strength increases of 8.96%, 10.65%, and 13.97%, respectively. Strength uniformity was further assessed using standard deviation analysis. Specimen U3 exhibited strength values ranging from 306 to 1350 kPa, with an overall standard deviation of 294.76 kPa. Specimen U4 showed a narrower range (392–1180 kPa) and a lower overall standard deviation of 216.07 kPa. Although the locations of maximum and minimum strengths were identical in both specimens, the consistently lower layer-wise standard deviations in U4 (161.01, 121.32, 157.97, and 187.98 kPa) relative to U3 (319.42, 201.82, 210.69, and 318.98 kPa) confirm the enhanced strength uniformity achieved using spiral-injection-one-extraction.

**Fig 8:**
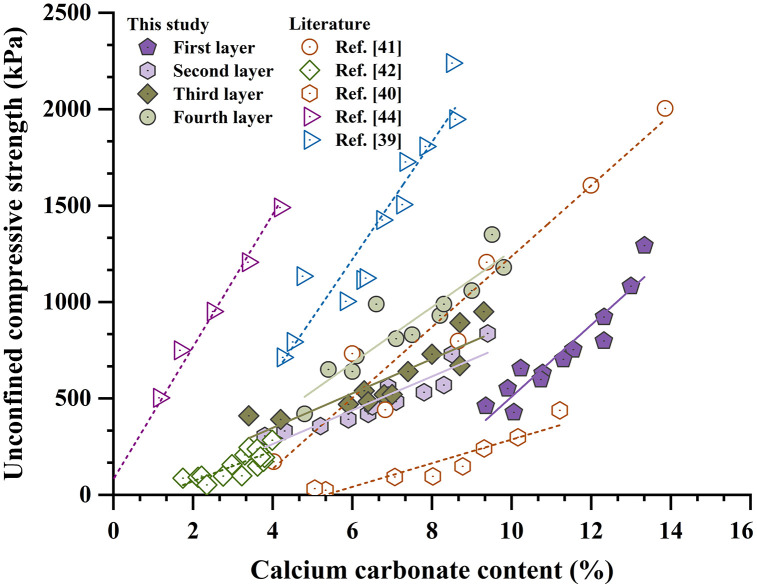
Relationships between calcium carbonate content and unconfined compressive strength.

**Fig 8** illustrates the relationship between calcium carbonate content and unconfined compressive strength for specimens U3 and U4, together with data from previous studies. The corresponding data are provided in S4 Table.An overall increasing trend in strength with calcium carbonate content is evident, consistent with existing literature [41–45]. However, a unique one-to-one relationship is not observed. At a calcium carbonate content of approximately 8%, unconfined compressive strength varied from 200 to 1700 kPa. This dispersion reflects the combined influence of particle size distribution, particle morphology, cementation uniformity, and carbonate crystal form [4,46]. In the present study, specimens from the bottom layers consistently exhibited higher strength at comparable calcium carbonate contents, suggesting more uniform cementation and possibly a greater proportion of mechanically stronger calcite crystals [24].

**Fig 9:**
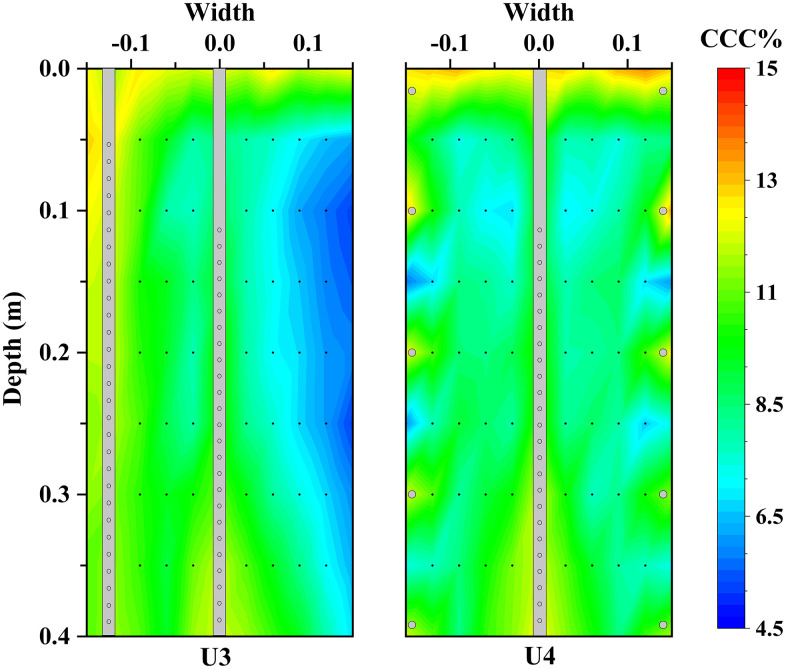
Spatial distribution of calcium carbonate precipitates.

**Fig 9** presents representative cross-sectional distributions of calcium carbonate in specimens U3 and U4 and the corresponding data are provided in S5 Table. Grey rectangles denote injection or extraction pipes, and black circles indicate perforation locations. In both specimens, calcium carbonate enrichment zones appear near the surface, base, and regions adjacent to injection and extraction points. Similar enrichment patterns were reported by van Paassen et al. [27] in large-scale MICP-treated sand foundations. Two mechanisms likely contribute to this behaviour: elevated concentrations of bacteria or cementation solution near injection and extraction points, and gravity-driven settling and accumulation of suspended carbonate crystals in regions of reduced flow velocity [40].

In specimen U3 (**Fig 9a**), pronounced carbonate enrichment is observed adjacent to the vertical injection pipes, extending vertically. Carbonate content decreases with depth near the pipe base, while regions distant from the injection pipes exhibit pronounced carbonate-deficient zones, indicating limited lateral spreading of grout and resulting non-uniform cementation. In contrast, specimen U4 (**Fig 9b**) exhibits a more homogeneous carbonate distribution without pronounced enrichment or weak zones. The helical arrangement of injection outlets promotes more uniform radial and vertical dispersion, facilitating more evenly distributed precipitation and bonding.

### 1.5 m sand column

The results above demonstrate that the spiral-injection-one-extraction configuration effectively mitigates the non-uniformity associated with conventional vertical pipes in 0.4 m-high sand columns. Its applicability at larger scale was further examined using a 1.5 m-high sand column (U5) treated with spiral-injection-one-extraction and the two-phase method.

**Fig 10:**
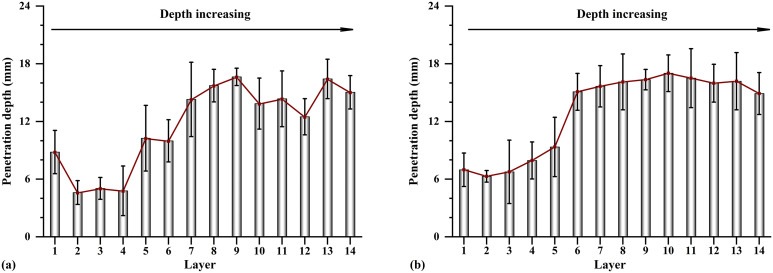
Distribution of surface penetration depth.

Surface penetration resistance was measured on two opposing half-column faces to minimise disturbance. The penetration depth profiles of both halves show similar depth-dependent trends (Fig 10 and S6 Table). Average penetration depths were relatively small in Layers 1–6, while values increased to approximately 14–16 mm below Layer 6, indicating reduced cementation strength at greater depths.

**Fig 11:**
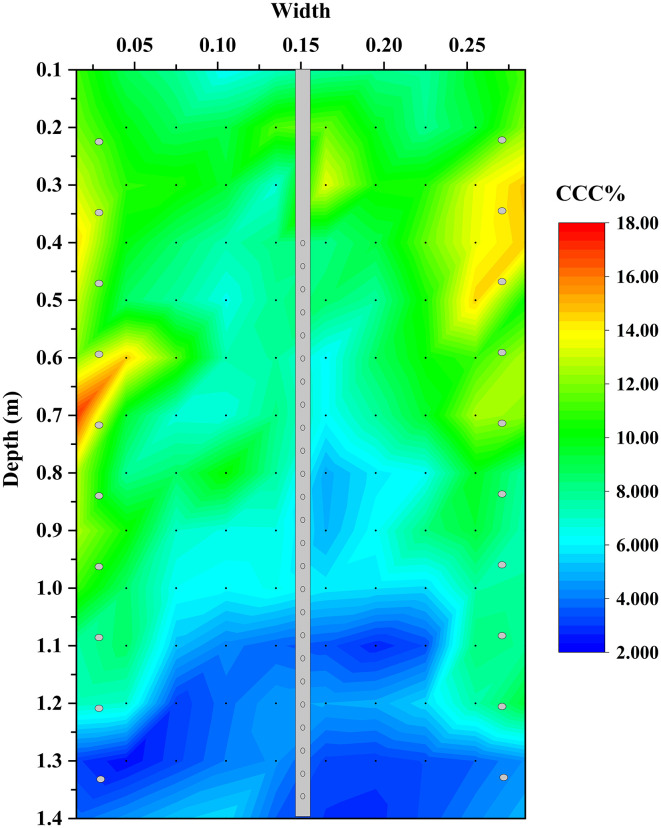
Distribution of calcium carbonate in 1.5 m sand column.

**Fig 11** presents the spatial distribution of calcium carbonate in specimen U5 and the corresponding data are provided in S7 Table. Along the depth direction, carbonate precipitation was concentrated primarily above a depth of 1.2 m near the injection pipes. In the radial direction, calcium carbonate content decreased with increasing distance from the pipes, consistent with the observations from specimen U4. Within the upper 0.6 m, the calcium carbonate distribution remained relatively uniform, whereas below this depth the calcium carbonate content near the extraction pipe decreased markedly relative to that near the injection region. These results indicate that the spiral-injection-one-extraction configuration remained effective in forming a large-scale cemented column, but its treatment uniformity decreased progressively with depth. This behaviour is attributed mainly to hydraulic energy losses associated with the small diameter and long flow path of the spiral hose. Under the constant injection pressure adopted in this study, solution transport efficiency at greater depths was reduced, thereby limiting the effective treatment depth. The present results therefore suggest that approximately 0.6 m was the depth range within which relatively uniform reinforcement could be achieved under the current pipe dimensions and pressure level, rather than a universal limiting depth for the spiral configuration itself.

**Fig 12:**
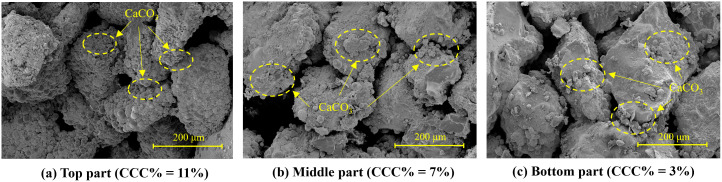
Scanning electron microscopy images of 1.5 m sand column at different heights.

Scanning electron microscopy was performed on samples extracted from depths of 0.1 m, 0.7 m, and 1.3 m (**Fig 3**), corresponding to calcium carbonate contents of approximately 11%, 7%, and 3%, respectively (**Fig 12**). At shallow depth (position a), sand particles are extensively coated by carbonate crystals, with interparticle voids partially filled and well-developed crystal bridges forming a continuous cemented structure. At intermediate depth (position b), fewer crystals are present, primarily adhering to particle surfaces, and interparticle bonding is less extensive. At greater depth (position c), only sparse carbonate crystals are observed at particle surfaces and contacts, resulting in weak cementation. Overall, the progressive reduction in crystal abundance and bonding strength with depth is consistent with the macroscopic calcium carbonate distribution shown in **Fig 11**.

## Discussions

### Effects of grouting pipe configuration on reinforcement uniformity and range

The spatial distribution of bacteria controls the homogeneity of calcium carbonate precipitation and thus the reinforcement efficiency of MICP in large-scale treatment. This distribution is closely related to bacterial migration and retention during grouting [33]. Grouting layout therefore directly affects solution transport and treatment uniformity [27]. The present results show that the one-pipe-injection-extraction configuration produces only a limited reinforcement zone. As shown in Figs 4, 5, effective cementation is mainly concentrated near the grouting pipe, and the effective reinforcement radius is about 0.09 m. This result is attributed to incomplete surface sealing and limited extraction-induced replacement, which confine fluid transport to a narrow region near the injection pipe. The results also confirm that the reinforcement range is strongly affected by pipe geometry and injection pressure [39].

Both the three-injection-one-extraction and spiral-injection-one-extraction configurations increase the effective reinforcement radius to about 0.15 m (Figs 6–8). However, their internal distributions differ. The vertical multi-pipe configuration produces relatively concentrated calcium carbonate enrichment zones and several weakly treated regions, whereas the spiral configuration produces smaller and more dispersed precipitation clusters, with more uniform distributions of both calcium carbonate content and unconfined compressive strength (Figs 6, 8–10). This improvement is mainly due to the spiral layout, which increases pipe-soil contact and outlet density [26], shortens the local transport distance in soil, and promotes more even solution redistribution toward the extraction pipe. These results show that pipe configuration affects not only the reinforcement range but also the internal uniformity of the cemented body.

### Trade-off between reinforcement uniformity and treatment depth

Although the spiral-injection-one-extraction configuration improves treatment uniformity, it also reduces treatment depth. Compared with conventional vertical pipes, the spiral hose has a smaller internal diameter and a longer internal flow path. These features increase hydraulic energy loss during fluid transport inside the pipe. Under the same injection pressure, less hydraulic energy remains available for deep solution migration. This effect is more obvious in the 1.5 m-high specimen. As shown in Figs 11, 12, relatively uniform reinforcement is mainly achieved within the upper 0.6 m, while the cementation degree decreases below this depth. This value should not be regarded as the fixed depth limit of the spiral configuration itself. Instead, it reflects the combined effect of the pipe dimensions, outlet size, and injection pressure of about 0.02 MPa used in this study.

These results indicate a trade-off between reinforcement uniformity and treatment depth. The spiral configuration improves uniformity by increasing outlet density and reducing the local flow distance in soil, but this benefit is accompanied by greater hydraulic losses inside the hose. In practical design, pipe number, pipe diameter, outlet size, helix pitch, injection pressure, and vertical spacing should be adjusted together according to the target treatment depth and reinforcement range.

### Uncertainty, limitations, and engineering implications

Several sources of uncertainty should be considered. First, local density differences may still exist in the sand column after layered compaction, and these differences may affect bacterial retention and solution flow. Second, preferential seepage paths may form during grouting and cause local enrichment or depletion of calcium carbonate precipitation. Third, specimen cutting, sampling, acid dissolution, and strength testing may introduce additional scatter into the measured calcium carbonate contents and unconfined compressive strengths. For compressive strength, the uncertainty was estimated from replicate UCS tests and expressed as the standard deviation of measured values. The coefficient of variation of UCS ranged from 2.32% to 5.41%, reflecting the combined effects of specimen heterogeneity, sample preparation, and testing variability. For calcium carbonate content, the uncertainty was evaluated from repeated acid-washing measurements and weighing precision, with the standard deviation ranging from 1.93% to 4.81%. These uncertainties indicate that, although local heterogeneity is unavoidable in biotreated sand, the overall trends in strength development and carbonate precipitation remain reliable. These factors partly explain the local fluctuations shown in Figs 5–12.

This study also has several limitations. Only one main pressure level is examined, and the effects of pipe diameter, outlet size, and helix pitch are not varied systematically. In addition, the effective reinforcement radius is defined using a calcium carbonate content threshold of 3%, which is suitable for the present tests but may not be directly applicable to other soils or treatment targets. The present results therefore identify reinforcement patterns and configuration effects, but do not establish a universal design standard.

Despite these limitations, the spiral-injection-one-extraction configuration shows clear engineering potential. It is suitable for cases where treatment uniformity is important or where conventional straight pipes are difficult to arrange, such as port yards, airport subgrades, and densely spaced foundation systems. The spiral layout is also flexible, because the reinforcement range can be adjusted through helix pitch, outlet size, discharge direction, and vertical spacing. With proper control of grouting parameters and field procedures, this configuration provides a practical way to improve the uniformity of field-scale MICP treatment.

## Conclusions

Large-scale MICP-treated sand column tests were conducted to evaluate the effects of grouting pipe configuration on calcium carbonate distribution, unconfined compressive strength, and treatment uniformity. Conventional vertical pipes and a newly proposed spiral-injection-one-extraction configuration were compared under different grouting strategies. Based on the experimental results, the following conclusions are drawn.

(1) Grouting pipe configuration controls the reinforcement range of large-scale MICP-treated sand columns. The one-pipe-injection-extraction configuration gives an effective reinforcement radius of about 0.09 m, whereas the three-injection-one-extraction and spiral-injection-one-extraction configurations increase it to about 0.15 m.
(2) The proposed spiral-injection-one-extraction configuration improves treatment uniformity more effectively than conventional vertical pipes. Although its reinforcement range is similar to that of the vertical multi-pipe configuration in this study, it produces a more uniform distribution of calcium carbonate content and unconfined compressive strength by reducing localized enrichment and weakly treated regions.
(3) For large-scale treated sand columns, unconfined compressive strength depends not only on calcium carbonate content, but also on grouting-induced flow behaviour. The clear depth dependence of the strength–calcium carbonate relationship shows that seepage path and local cementation structure strongly affect reinforcement efficiency at scale.
(4) The spiral configuration improves uniformity but limits treatment depth under the present test conditions. In the 1.5 m-high specimen, relatively uniform reinforcement is mainly achieved within the upper 0.6 m, indicating that pipe diameter, outlet size, and injection pressure must be jointly optimised for deeper treatment.

## Supporting information

S1 Table Raw data corresponding to Fig 5. (DOCX)

S2 Table Raw data corresponding to Fig 6. (DOCX)

S3 Table Raw data corresponding to Fig 7. (DOCX)

S4 Table Raw data corresponding to Fig 8. (DOCX)

S5 Table Raw data corresponding to Fig 9. (DOCX)

S6 Table Raw data corresponding to Fig 10. (DOCX)

S7 Table Raw data corresponding to Fig 11. (DOCX)

## References

[1] Zhang Y, Hu X, Wang Y, Jiang N. A critical review of biomineralization in environmental geotechnics: applications, trends, and perspectives. Biogeotechnics. 2023;1(1):100003. doi: 10.1016/j.bgtech.2023.100003

[2] He J, Liu Y, Liu L, Yan B, Li L, Meng H, et al. Recent development on optimization of bio-cementation for soil stabilization and wind erosion control. Biogeotechnics. 2023;1(2):100022. doi: 10.1016/j.bgtech.2023.100022

[3] Umar M, Kassim KA, Ping Chiet KT. Biological process of soil improvement in civil engineering: a review. J Rock Mech Geotech Eng. 2016;8(5):767–74. doi: 10.1016/j.jrmge.2016.02.004

[4] Xiao Y, He X, Zaman M, Ma GL, Zhao C. Review of strength improvements of biocemented soils. Int J Geomech. 2022;22(11):03122001. doi: 10.1061/(Asce)Gm.1943-5622.0002565

[5] He X, Gu L, Zhang C, Yang Y, Li X, Ye L, et al. Use of MICP with active bioslurry for restoration of fragmented ceramic artifacts: Mechanical and fracture behaviors study. Case Stud Construct Mater. 2025;22:e04626. doi: 10.1016/j.cscm.2025.e04626

[6] Mitchell JK, Santamarina JC. Biological considerations in geotechnical engineering. J Geotech Geoenviron Eng. 2005;131(10):1222–33. doi: 10.1061/(Asce)1090-0241(2005)131:10(1222

[7] Jiang N, Wang Y, Chu J, Kawasaki S, Tang C, Cheng L, et al. Bio‐mediated soil improvement: An introspection into processes, materials, characterization and applications. Soil Use Manage. 2021;38(1):68–93. doi: 10.1111/sum.12736

[8] El Mountassir G, Minto JM, van Paassen LA, Salifu E, Lunn RJ. Applications of microbial processes in geotechnical engineering. Adv Appl Microbiol. 2018;104:39–91. doi: 10.1016/bs.aambs.2018.05.001 30143252

[9] Xiao Y, He X, Ma G, Zhao C, Chu J, Liu H. Biomineralization and mineralization using microfluidics: a comparison study. J Rock Mech Geotech Eng. 2024;16(2):661–9. doi: 10.1016/j.jrmge.2023.03.019

[10] Lai H, Ding X, Cui M, Zheng J, Chu J, Chen Z. Factors affecting the effectiveness of biocementation of soil. Biogeotechnics. 2024;2(3):100087. doi: 10.1016/j.bgtech.2024.100087

[11] DeJong JT, Mortensen BM, Martinez BC, Nelson DC. Bio-mediated soil improvement. Ecol Eng. 2010;36(2):197–210. doi: 10.1016/j.ecoleng.2008.12.029

[12] Xiao Y, He X, Stuedlein AW, Chu J, Evans TM, van Paassen LA. Crystal growth of micp through microfluidic chip tests. J Geotech Geoenviron Eng. 2022;148(5):06022002. doi: 10.1061/(Asce)Gt.1943-5606.0002756

[13] Ma GL, He X, Xiao Y, Chu J, Liu HL, Stuedlein AW. Spatiotemporal evolution of biomineralization in heterogeneous pore structure. Can Geotech J. 2024;61(3):447–68. doi: 10.1139/cgj-2022-0496447

[14] Zhao C, Xiao Y, He X, Cui H, Liu HL. Eicp-enhanced fracture healing: bridging microfluidic observations and macroscale applications. Geotechnique. 2025;75(10):1269–81. doi: 10.1680/jgeot.24.01217

[15] Xiao Y, Yan J, Wu HR, Zaman M. Tensile strength and fracture of interparticle micp bonds. Int J Geomech. 2024;24(10):06024019. doi: 10.1061/Ijgnai.Gmeng-10233

[16] Xiao Y, Xiao W, Wu H, Liu Y, Liu H. Fracture of interparticle micp bonds under compression. Int J Geomech. 2023;23(3):04022316. doi: 10.1061/ijgnai.Gmeng-8282

[17] Lin H, Suleiman MT, Brown DG. Investigation of pore-scale CaCO3 distributions and their effects on stiffness and permeability of sands treated by microbially induced carbonate precipitation (MICP). Soils Found. 2020;60(4):944–61. doi: 10.1016/j.sandf.2020.07.003

[18] Fu TZ, Haigh SK. Biocementation of a well-graded gravelly soil and macromechanical characterization. J Geotech Geoenviron Eng. 2024;150(8):04024061.

[19] Lin H, Dong Y, Park JS, Montoya BM. Cementation stress characteristic curve for sands treated by microbially induced carbonate precipitation. J Geotech Geoenviron Eng. 2023;149(12):04023115. doi: 10.1061/Jggefk.Gteng-11403

[20] Zamani A, Montoya BM. Undrained cyclic response of silty sands improved by microbial induced calcium carbonate precipitation. Soil Dyn Earthq Eng. 2019;120:436–48. doi: 10.1016/j.soildyn.2019.01.010

[21] Zamani A, Montoya BM, Gabr MA. Investigating challenges of in situ delivery of microbial-induced calcium carbonate precipitation (micp) in fine-grain sands and silty sand. Can Geotech J. 2019;56(12):1889–900. doi: 10.1139/cgj-2018-0551

[22] Montoya BM, Do J, Gabr MA. Distribution and properties of microbially induced carbonate precipitation in underwater sand bed. J Geotech Geoenviron Eng. 2021;147(10):04021098. doi: 10.1061/(Asce)Gt.1943-5606.0002607

[23] Zamani A, Xiao P, Baumer T, Carey TJ, Sawyer B, DeJong JT, et al. Mitigation of liquefaction triggering and foundation settlement by micp treatment. J Geotech Geoenviron Eng. 2021;147(10):04021099. doi: 10.1061/(Asce)Gt.1943-5606.0002596

[24] Sang GJ, Lunn RJ, El Mountassir G, Minto JM. Meter-scale micp improvement of medium graded very gravelly sands: lab measurement, transport modelling, mechanical and microstructural analysis. Eng Geol. 2023;324:107275. doi: 10.1016/j.enggeo.2023.107275

[25] Sang G, Lunn RJ, El Mountassir G, Minto JM, McLachlan E, Bradley D. Improving non-uniform gravelly sand using microbially induced carbonate precipitation: An outdoor cubic-meter scale trial by engineering contractors. Eng Geol. 2024;343:107791. doi: 10.1016/j.enggeo.2024.107791

[26] Whiffin VS, van Paassen LA, Harkes MP. Microbial carbonate precipitation as a soil improvement technique. Geomicrobiol J. 2007;24(5):417–23. doi: 10.1080/01490450701436505

[27] van Paassen LA, Ghose R, van der Linden TJM, van der Star WRL, van Loosdrecht MCM. Quantifying biomediated ground improvement by ureolysis: large-scale biogrout experiment. J Geotech Geoenviron Eng. 2010;136(12):1721–8. doi: 10.1061/(asce)gt.1943-5606.0000382

[28] Sharma M, Satyam N, Reddy KR. Large-scale spatial characterization and liquefaction resistance of sand by hybrid bacteria induced biocementation. Eng Geol. 2022;302:106635.

[29] Gomez MG, Anderson CM, Graddy CMR, DeJong JT, Nelson DC, Ginn TR. Large-scale comparison of bioaugmentation and biostimulation approaches for biocementation of sands. J Geotech Geoenviron Eng. 2017;143(5):04016124. doi: 10.1061/(Asce)Gt.1943-5606.0001640

[30] Nassar MK, Gurung D, Bastani M, Ginn TR, Shafei B, Gomez MG, et al. Large‐scale experiments in microbially induced calcite precipitation (MICP): reactive transport model development and prediction. Water Resourc Res. 2018;54(1):480–500. doi: 10.1002/2017wr021488

[31] Martinez BC, DeJong JT, Ginn TR, Montoya BM, Barkouki TH, Hunt C. Experimental optimization of microbial-induced carbonate precipitation for soil improvement. J Geotech Geoenviron Eng. 2013;139(4):587–98. doi: 10.1061/(Asce)Gt.1943-5606.0000787

[32] San Pablo ACM, Lee M, Graddy CMR, Kolbus CM, Khan M, Zamani A. Meter-scale biocementation experiments to advance process control and reduce impacts: examining spatial control, ammonium by-product removal, and chemical reductions. J Geotech Geoenviron Eng. 2020;146(11):04020125. doi: 10.1061/(asce)gt.1943-5606.0002377

[33] Zheng JJ, Lai HJ, Cui MJ, Ding XZ, Weng YJ, Zhang JW. Bio-grouting technologies for enhancing uniformity of biocementation: a review. Biogetech. 2023;1(3):100033. doi: 10.1016/j.bgtech.2023.100033

[34] Xiao Y, Wu BY, Shi JQ, Wang L, Liu HL. Acoustic emission of biocemented calcareous sand base. Int J Geomech. 2023;23(9):04023153. doi: 10.1061/Ijgnai.Gmeng-8817

[35] Almajed A, Tirkolaei HK, Kavazanjian E. Baseline investigation on enzyme-induced calcium carbonate precipitation. J Geotech Geoenviron Eng. 2018;144(11):04018081. doi: 10.1061/(Asce)Gt.1943-5606.0001973

[36] Lai H-J, Cui M-J, Chu J. Effect of pH on soil improvement using one-phase-low-pH MICP or EICP biocementation method. Acta Geotech. 2022;18(6):3259–72. doi: 10.1007/s11440-022-01759-3

[37] Xiao Y, He X, Evans TM, Stuedlein AW, Liu HL. Unconfined compressive and splitting tensile strength of basalt fiber–reinforced biocemented sand. J Geotech Geoenviron Eng. 2019;145(9):04019048. doi: 10.1061/(ASCE)GT.1943-5606.0002108

[38] Liu H, Wei Z, He X, Wang C. Effect of skim milk powder and injection method on efficiency and uniformity of bio-treated 0.5 m-scale sand column. Sci China Technol Sci. 2025;68(5). doi: 10.1007/s11431-024-2898-9

[39] Martin KK, Tirkolaei HK, Kavazanjian E. Field-scale eicp biocemented columns for ground improvement. J Geotech Geoenviron Eng. 2024;150(8):05024006. doi: 10.1061/Jggefk.Gteng-11635

[40] Wu S, Li B, Chu J. Large-scale model tests of biogrouting for sand and rock. Proc Inst Civil Eng - Ground Improve. 2023;176(1):23–32. doi: 10.1680/jgrim.18.00074

[41] Ma GL, He X, Jiang X, Liu HL, Chu J, Xiao Y. Strength and permeability of bentonite-assisted biocemented coarse sand. Can Geotech J. 2021;58(7):969–81. doi: 10.1139/cgj-2020-0045

[42] Cheng L, Shahin MA, Cord-Ruwisch R. Bio-cementation of sandy soil using microbially induced carbonate precipitation for marine environments. Geotechnique. 2014;64(12):1010–3. doi: 10.1680/geot.14.T.025

[43] Cheng L, Cord-Ruwisch R, Shahin MA. Cementation of sand soil by microbially induced calcite precipitation at various degrees of saturation. Can Geotech J. 2013;50(1):81–90. doi: 10.1139/cgj-2012-0023

[44] Wu C, Chu J, Cheng L, Wu S. Biogrouting of aggregates using premixed injection method with or without pH adjustment. J Mater Civ Eng. 2019;31(9). doi: 10.1061/(asce)mt.1943-5533.0002874

[45] Stabnikov V, Chu J, Ivanov V, Li Y. Halotolerant, alkaliphilic urease-producing bacteria from different climate zones and their application for biocementation of sand. World J Microbiol Biotechnol. 2013;29(8):1453–60. doi: 10.1007/s11274-013-1309-1 23529354

[46] Zhang Z, Wang K, Wu S, Chu J. Amorphous calcium carbonate (ACC) cement for ground improvement. Acta Geotech. 2025;20(6):2557–71. doi: 10.1007/s11440-025-02543-9

