# Supplementary material for: Large-scale experimental investigation of biotreated sand column using different grouting pipe configurations
Source: PLoS One. 2026 May 26;21(5):e0349797. doi: 10.1371/journal.pone.0349797 (PMC13210374; doi:10.1371/journal.pone.0349797)
Supplement: S1 Table — (DOCX) [file pone.0349797.s001.docx]

**S1 Table. Raw data corresponding to Fig 5**

| U1 | | | U2 | | |
| --- | --- | --- | --- | --- | --- |
| Width (m) | Depth  (m) | Calcium Carbonate content  (%) | Width (m) | Depth  (m) | Calcium Carbonate content  (%) |
| 0.03 | 0 | 7.5 | 0.03 | 0 | 7.3 |
| 0.03 | 0.05 | 6.94 | 0.03 | 0.05 | 6.178 |
| 0.03 | 0.1 | 5.7 | 0.03 | 0.1 | 6.645 |
| 0.03 | 0.15 | 5.26 | 0.03 | 0.15 | 5.22 |
| 0.03 | 0.2 | 4.19 | 0.03 | 0.2 | 5.409 |
| 0.03 | 0.25 | 4.68 | 0.03 | 0.25 | 5.875 |
| 0.03 | 0.3 | 5.39 | 0.03 | 0.3 | 7.288 |
| 0.03 | 0.35 | 4.64 | 0.03 | 0.35 | 8.3 |
| 0.03 | 0.4 | 6.52 | 0.03 | 0.4 | 7.72 |
| 0.06 | 0 | 6.9 | 0.06 | 0 | 6.4 |
| 0.06 | 0.05 | 4.078 | 0.06 | 0.05 | 5.34 |
| 0.06 | 0.1 | 4.188 | 0.06 | 0.1 | 4.3 |
| 0.06 | 0.15 | 3.876 | 0.06 | 0.15 | 3.83 |
| 0.06 | 0.2 | 4.467 | 0.06 | 0.2 | 3.13 |
| 0.06 | 0.25 | 3.36 | 0.06 | 0.25 | 3.1285 |
| 0.06 | 0.3 | 4.14 | 0.06 | 0.3 | 3.373 |
| 0.06 | 0.35 | 4.29 | 0.06 | 0.35 | 4.4319 |
| 0.06 | 0.4 | 5.64 | 0.06 | 0.4 | 6.3 |
| 0.09 | 0 | 2.8 | 0.09 | 0 | 6.7 |
| 0.09 | 0.05 | 2.55 | 0.09 | 0.05 | 4.158 |
| 0.09 | 0.1 | 1.52 | 0.09 | 0.1 | 2.64 |
| 0.09 | 0.15 | 2.1 | 0.09 | 0.15 | 1.7 |
| 0.09 | 0.2 | 1.817 | 0.09 | 0.2 | 2.59 |
| 0.09 | 0.25 | 1.969 | 0.09 | 0.25 | 2.396 |
| 0.09 | 0.3 | 2.081 | 0.09 | 0.3 | 2.424 |
| 0.09 | 0.35 | 2.567 | 0.09 | 0.35 | 3.289 |
| 0.09 | 0.4 | 2.92 | 0.09 | 0.4 | 3.4 |
| 0.12 | 0 | 2.5 | 0.12 | 0 | 5.6 |
| 0.12 | 0.05 | 2.33 | 0.12 | 0.05 | 1.786 |
| 0.12 | 0.1 | 1.11 | 0.12 | 0.1 | 1.05 |
| 0.12 | 0.15 | 1.176 | 0.12 | 0.15 | 1.42 |
| 0.12 | 0.2 | 1.236 | 0.12 | 0.2 | 1.049 |
| 0.12 | 0.25 | 1.439 | 0.12 | 0.25 | 0.977 |
| 0.12 | 0.3 | 1.876 | 0.12 | 0.3 | 1.058 |
| 0.12 | 0.35 | 1.39 | 0.12 | 0.35 | 2.543 |
| 0.12 | 0.4 | 1.83 | 0.12 | 0.4 | 3.619 |
| 0.15 | 0 | 1.5 | 0.15 | 0 | 4.2 |
| 0.15 | 0.05 | 1.03 | 0.15 | 0.05 | 1.1 |
| 0.15 | 0.1 | 0.9 | 0.15 | 0.1 | 0.8 |
| 0.15 | 0.15 | 0.7 | 0.15 | 0.15 | 0.9 |
| 0.15 | 0.2 | 0.6 | 0.15 | 0.2 | 0.5 |
| 0.15 | 0.25 | 0.75 | 0.15 | 0.25 | 0.4 |
| 0.15 | 0.3 | 0.89 | 0.15 | 0.3 | 0.7 |
| 0.15 | 0.35 | 1 | 0.15 | 0.35 | 0.65 |
| 0.15 | 0.4 | 1.1 | 0.15 | 0.4 | 1.1 |
