# Supplementary material for: Large-scale experimental investigation of biotreated sand column using different grouting pipe configurations
Source: PLoS One. 2026 May 26;21(5):e0349797. doi: 10.1371/journal.pone.0349797 (PMC13210374; doi:10.1371/journal.pone.0349797)
Supplement: S2 Table — (DOCX) [file pone.0349797.s002.docx]

**S2 Table. Raw data corresponding to Fig 6**

| Figure 6(a) | | |
| --- | --- | --- |
| Sample ID | U3 Calcium carbonate content (%) | U4 Calcium carbonate content (%) |
| A1 | 12.995 | 12.32 |
| A2 | 13.334 | 11.55 |
| A3 | 12.317 | 10.78 |
| A4 | 11.3 | 10.23 |
| A5 | 10.735 | 9.9 |
| A6 | 10.057 | 9.35 |
| Figure 6(b) | | |
| Sample ID | U3 Calcium carbonate content (%) | U4 Calcium carbonate content (%) |
| B1 | 9.4 | 8.5 |
| B2 | 8.3 | 7.8 |
| B3 | 6.5 | 6.9 |
| B4 | 5.2 | 7.1 |
| B5 | 4.3 | 6.4 |
| B6 | 3.8 | 5.9 |
| Figure 6(c) | | |
| Sample ID | U3 Calcium carbonate content (%) | U4 Calcium carbonate content (%) |
| C1 | 9.3 | 8.7 |
| C2 | 8.7 | 8 |
| C3 | 7 | 7.4 |
| C4 | 6.4 | 6.8 |
| C5 | 5.2 | 6.3 |
| C6 | 3.4 | 5.9 |
| Figure 6(d) | | |
| Sample ID | U3 Calcium carbonate content (%) | U4 Calcium carbonate content (%) |
| D1 | 9.51 | 9.8 |
| D2 | 8.3 | 9 |
| D3 | 7.1 | 8.2 |
| D4 | 6.1 | 7.5 |
| D5 | 5.4 | 6.6 |
| D6 | 4.8 | 6 |
