# Supplementary material for: Large-scale experimental investigation of biotreated sand column using different grouting pipe configurations
Source: PLoS One. 2026 May 26;21(5):e0349797. doi: 10.1371/journal.pone.0349797 (PMC13210374; doi:10.1371/journal.pone.0349797)
Supplement: S3 Table — (DOCX) [file pone.0349797.s003.docx]

**S3 Table. Raw data corresponding to Fig 7**

| Figure 7(a) | | |
| --- | --- | --- |
| Sample ID | U3 Unconfined compressive strength (kPa) | U4 Unconfined compressive strength (kPa) |
| A1 | 1083 | 921.5 |
| A2 | 1293 | 753.35 |
| A3 | 798 | 630.8 |
| A4 | 703 | 655.5 |
| A5 | 598.5 | 551 |
| A6 | 427.5 | 460.5 |
| Figure 7(b) | | |
| Sample ID | U3 Calcium carbonate content (%) | U4 Calcium carbonate content (%) |
| B1 | 837.5 | 730 |
| B2 | 569.5 | 532 |
| B3 | 459 | 560 |
| B4 | 357 | 483 |
| B5 | 331.5 | 420 |
| B6 | 306 | 392 |
| Figure 7(c) | | |
| Sample ID | U3 Calcium carbonate content (%) | U4 Calcium carbonate content (%) |
| C1 | 950 | 892.5 |
| C2 | 670.5 | 730 |
| C3 | 521 | 640 |
| C4 | 483 | 520 |
| C5 | 391 | 540 |
| C6 | 410.5 | 470 |
| Figure 7(d) | | |
| Sample ID | U3 Calcium carbonate content (%) | U4 Calcium carbonate content (%) |
| D1 | 1350 | 1180 |
| D2 | 990 | 1060 |
| D3 | 810 | 930 |
| D4 | 720 | 830 |
| D5 | 650 | 990 |
| D6 | 420 | 640 |
