# Supplementary material for: Large-scale experimental investigation of biotreated sand column using different grouting pipe configurations
Source: PLoS One. 2026 May 26;21(5):e0349797. doi: 10.1371/journal.pone.0349797 (PMC13210374; doi:10.1371/journal.pone.0349797)
Supplement: S4 Table — (DOCX) [file pone.0349797.s004.docx]

**S4 Table. Raw data corresponding to Fig 8**

| First layer | |
| --- | --- |
| Calcium carbonate content (%) | Unconfined compressive strength (kPa) |
| 12.32 | 921.5 |
| 11.55 | 753.35 |
| 10.78 | 630.8 |
| 10.23 | 655.5 |
| 9.9 | 551 |
| 9.35 | 460.5 |
| 12.995 | 1083 |
| 13.334 | 1293 |
| 12.317 | 798 |
| 11.3 | 703 |
| 10.735 | 598.5 |
| 10.057 | 427.5 |
| Second layer | |
| Calcium carbonate content (%) | Unconfined compressive strength (kPa) |
| 8.5 | 730 |
| 7.8 | 532 |
| 6.9 | 560 |
| 7.1 | 483 |
| 6.4 | 420 |
| 5.9 | 392 |
| 9.4 | 837.5 |
| 8.3 | 569.5 |
| 6.5 | 459 |
| 5.2 | 357 |
| 4.3 | 331.5 |
| 3.8 | 306 |
| Third layer | |
| Calcium carbonate content (%) | Unconfined compressive strength (kPa) |
| 8.7 | 892.5 |
| 8 | 730 |
| 7.4 | 640 |
| 6.8 | 520 |
| 6.3 | 540 |
| 5.9 | 470 |
| 9.3 | 950 |
| 8.7 | 670.5 |
| 7 | 521 |
| 6.4 | 483 |
| 4.2 | 391 |
| 3.4 | 410.5 |
| Fourth layer | |
| Calcium carbonate content (%) | Unconfined compressive strength (kPa) |
| 9.8 | 1180 |
| 9 | 1060 |
| 8.2 | 930 |
| 7.5 | 830 |
| 6.6 | 990 |
| 6 | 640 |
| 9.51 | 1350 |
| 8.3 | 990 |
| 7.1 | 810 |
| 6.1 | 720 |
| 5.4 | 650 |
| 4.8 | 420 |
| Ref. [41] | |
| Calcium carbonate content (%) | Unconfined compressive strength (kPa) |
| 4.0263 | 172.414 |
| 6.82818 | 441.81 |
| 5.99665 | 732.759 |
| 8.65759 | 797.414 |
| 9.37112 | 1206.9 |
| 11.9913 | 1605.6 |
| 13.8561 | 2004.31 |
| Ref. [42] | |
| Calcium carbonate content (%) | Unconfined compressive strength (kPa) |
| 1.74487 | 86.9822 |
| 2.1261 | 91.1243 |
| 2.21408 | 97.3373 |
| 2.34604 | 51.7751 |
| 2.7566 | 97.3373 |
| 3.22581 | 99.4083 |
| 2.97654 | 157.396 |
| 3.29912 | 207.101 |
| 3.40176 | 246.45 |
| 3.60704 | 238.166 |
| 3.6217 | 149.112 |
| 3.76833 | 173.964 |
| 3.79765 | 194.675 |
| 3.69501 | 196.746 |
| 3.98827 | 283.728 |
| Ref. [40] | |
| Calcium carbonate content (%) | Unconfined compressive strength (kPa) |
| 5.32966 | 24.6575 |
| 7.06637 | 93.1507 |
| 9.31052 | 241.096 |
| 10.1605 | 298.63 |
| 11.2106 | 438.356 |
| 5.05615 | 32.8767 |
| 8.02185 | 95.8904 |
| 8.76974 | 147.945 |
| Ref. [39] | |
| Calcium carbonate content (%) | Unconfined compressive strength (kPa) |
| 4.25 | 712.851 |
| 4.5 | 793.173 |
| 5.83333 | 1004.02 |
| 6.25 | 1114.46 |
| 6.33333 | 1124.5 |
| 4.75 | 1134.54 |
| 6.75 | 1425.7 |
| 7.25 | 1506.02 |
| 7.33333 | 1726.91 |
| 7.83333 | 1807.23 |
| 8.58333 | 1947.79 |
| 8.5 | 2238.96 |
| Ref. [44] | |
| Calcium carbonate content (%) | Unconfined compressive strength (kPa) |
| 1.14262 | 502.857 |
| 1.66691 | 749.714 |
| 2.49299 | 950.857 |
| 3.37492 | 1206.86 |
| 4.18121 | 1490.29 |
