# Supplementary material for: Large-scale experimental investigation of biotreated sand column using different grouting pipe configurations
Source: PLoS One. 2026 May 26;21(5):e0349797. doi: 10.1371/journal.pone.0349797 (PMC13210374; doi:10.1371/journal.pone.0349797)
Supplement: S5 Table — (DOCX) [file pone.0349797.s005.docx]

**S5 Table. Raw data corresponding to Fig 9**

| U3 | | | U4 | | |
| --- | --- | --- | --- | --- | --- |
| Width (m) | Depth  (m) | Calcium Carbonate content  (%) | Width (m) | Depth  (m) | Calcium Carbonate content  (%) |
| -0.15 | 0 | 11.894 | -0.15 | 0 | 11.8272 |
| -0.12 | 0 | 10.9725 | -0.12 | 0 | 12.048 |
| -0.09 | 0 | 12.141 | -0.09 | 0 | 12.2688 |
| -0.06 | 0 | 11.6185 | -0.06 | 0 | 11.7408 |
| -0.03 | 0 | 11.4 | -0.03 | 0 | 11.52 |
| 0 | 0 | 11.7325 | 0 | 0 | 11.856 |
| 0.03 | 0 | 11.305 | 0.03 | 0 | 12 |
| 0.06 | 0 | 12.255 | 0.06 | 0 | 11.424 |
| 0.09 | 0 | 11.21 | 0.09 | 0 | 12.288 |
| 0.12 | 0 | 11.59 | 0.12 | 0 | 12.672 |
| 0.15 | 0 | 11.875 | 0.15 | 0 | 12 |
| -0.15 | 0.05 | 12.35 | -0.15 | 0.05 | 9.6 |
| -0.12 | 0.05 | 11.875 | -0.12 | 0.05 | 8.448 |
| -0.09 | 0.05 | 10.45 | -0.09 | 0.05 | 7.2 |
| -0.06 | 0.05 | 9.405 | -0.06 | 0.05 | 7.584 |
| -0.03 | 0.05 | 7.98 | -0.03 | 0.05 | 8.064 |
| 0 | 0.05 | 8.36 | 0 | 0.05 | 9.6 |
| 0.03 | 0.05 | 7.923 | 0.03 | 0.05 | 7.6224 |
| 0.06 | 0.05 | 7.5791 | 0.06 | 0.05 | 7.75488 |
| 0.09 | 0.05 | 6.9825 | 0.09 | 0.05 | 7.248 |
| 0.12 | 0.05 | 6.4885 | 0.12 | 0.05 | 7.9968 |
| 0.15 | 0.05 | 6.175 | 0.15 | 0.05 | 8.16 |
| -0.15 | 0.1 | 11.875 | -0.15 | 0.1 | 12 |
| -0.12 | 0.1 | 11.3905 | -0.12 | 0.1 | 9.576 |
| -0.09 | 0.1 | 10.355 | -0.09 | 0.1 | 7.6608 |
| -0.06 | 0.1 | 7.98 | -0.06 | 0.1 | 6.9312 |
| -0.03 | 0.1 | 7.695 | -0.03 | 0.1 | 6.6576 |
| 0 | 0.1 | 8.74 | 0 | 0.1 | 9.216 |
| 0.03 | 0.1 | 7.885 | 0.03 | 0.1 | 6.84 |
| 0.06 | 0.1 | 7.106 | 0.06 | 0.1 | 7.18656 |
| 0.09 | 0.1 | 6.194 | 0.09 | 0.1 | 7.77024 |
| 0.12 | 0.1 | 5.71045 | 0.12 | 0.1 | 9.22032 |
| 0.15 | 0.1 | 5.225 | 0.15 | 0.1 | 12.384 |
| -0.15 | 0.15 | 11.4 | -0.15 | 0.15 | 5.68608 |
| -0.12 | 0.15 | 11.305 | -0.12 | 0.15 | 6.61152 |
| -0.09 | 0.15 | 9.785 | -0.09 | 0.15 | 8.19456 |
| -0.06 | 0.15 | 9.31 | -0.06 | 0.15 | 8.00832 |
| -0.03 | 0.15 | 8.36 | -0.03 | 0.15 | 7.47754 |
| 0 | 0.15 | 9.31 | 0 | 0.15 | 9.12576 |
| 0.03 | 0.15 | 7.847 | 0.03 | 0.15 | 7.69171 |
| 0.06 | 0.15 | 7.22 | 0.06 | 0.15 | 8.26533 |
| 0.09 | 0.15 | 6.5645 | 0.09 | 0.15 | 8.47392 |
| 0.12 | 0.15 | 5.8672 | 0.12 | 0.15 | 6.68229 |
| 0.15 | 0.15 | 5.51 | 0.15 | 0.15 | 6.04118 |
| -0.15 | 0.2 | 11.21 | -0.15 | 0.2 | 11.328 |
| -0.12 | 0.2 | 11.305 | -0.12 | 0.2 | 9.9696 |
| -0.09 | 0.2 | 9.975 | -0.09 | 0.2 | 8.28768 |
| -0.06 | 0.2 | 9.215 | -0.06 | 0.2 | 8.19456 |
| -0.03 | 0.2 | 7.885 | -0.03 | 0.2 | 8.53066 |
| 0 | 0.2 | 9.975 | 0 | 0.2 | 9.7776 |
| 0.03 | 0.2 | 7.9705 | 0.03 | 0.2 | 7.81277 |
| 0.06 | 0.2 | 7.09365 | 0.06 | 0.2 | 7.88447 |
| 0.09 | 0.2 | 6.76115 | 0.09 | 0.2 | 8.21039 |
| 0.12 | 0.2 | 6.1142 | 0.12 | 0.2 | 9.87763 |
| 0.15 | 0.2 | 5.89 | 0.15 | 0.2 | 11.424 |
| -0.15 | 0.2 | 11.21 | -0.15 | 0.25 | 5.9472 |
| -0.15 | 0.25 | 10.735 | -0.12 | 0.25 | 7.6608 |
| -0.12 | 0.25 | 11.115 | -0.09 | 0.25 | 8.8704 |
| -0.09 | 0.25 | 10.26 | -0.06 | 0.25 | 8.3664 |
| -0.06 | 0.25 | 8.835 | -0.03 | 0.25 | 7.9632 |
| -0.03 | 0.25 | 8.075 | 0 | 0.25 | 10.08 |
| 0 | 0.25 | 9.975 | 0.03 | 0.25 | 7.94304 |
| 0.03 | 0.25 | 8.246 | 0.06 | 0.25 | 8.42688 |
| 0.06 | 0.25 | 7.562 | 0.09 | 0.25 | 7.83115 |
| 0.09 | 0.25 | 6.71555 | 0.12 | 0.25 | 6.49051 |
| 0.12 | 0.25 | 6.11705 | 0.15 | 0.25 | 7.2 |
| 0.15 | 0.25 | 5.225 | -0.15 | 0.25 | 5.9472 |
| -0.15 | 0.3 | 10.925 | -0.15 | 0.3 | 11.04 |
| -0.12 | 0.3 | 10.64 | -0.12 | 0.3 | 10.08 |
| -0.09 | 0.3 | 9.975 | -0.09 | 0.3 | 7.968 |
| -0.06 | 0.3 | 9.31 | -0.06 | 0.3 | 8.832 |
| -0.03 | 0.3 | 9.785 | -0.03 | 0.3 | 9.408 |
| 0 | 0.3 | 10.45 | 0 | 0.3 | 11.04 |
| 0.03 | 0.3 | 9.3955 | 0.03 | 0.3 | 9.3984 |
| 0.06 | 0.3 | 8.113 | 0.06 | 0.3 | 7.59744 |
| 0.09 | 0.3 | 7.4195 | 0.09 | 0.3 | 8.04576 |
| 0.12 | 0.3 | 6.7222 | 0.12 | 0.3 | 9.48096 |
| 0.15 | 0.3 | 5.985 | 0.15 | 0.3 | 10.464 |
| -0.15 | 0.35 | 10.355 | -0.15 | 0.35 | 7.296 |
| -0.12 | 0.35 | 10.83 | -0.12 | 0.35 | 7.584 |
| -0.09 | 0.35 | 10.07 | -0.09 | 0.35 | 8.256 |
| -0.06 | 0.35 | 9.025 | -0.06 | 0.35 | 9.408 |
| -0.03 | 0.35 | 10.545 | -0.03 | 0.35 | 10.368 |
| 0 | 0.35 | 11.305 | 0 | 0.35 | 11.04 |
| 0.03 | 0.35 | 10.355 | 0.03 | 0.35 | 10.08 |
| 0.06 | 0.35 | 9.31 | 0.06 | 0.35 | 8.928 |
| 0.09 | 0.35 | 8.265 | 0.09 | 0.35 | 7.872 |
| 0.12 | 0.35 | 7.1345 | 0.12 | 0.35 | 7.584 |
| 0.15 | 0.35 | 6.365 | 0.15 | 0.35 | 7.2 |
| -0.15 | 0.4 | 10.355 | -0.15 | 0.4 | 11.04 |
| -0.12 | 0.4 | 10.925 | -0.12 | 0.4 | 10.08 |
| -0.09 | 0.4 | 10.355 | -0.09 | 0.4 | 8.16 |
| -0.06 | 0.4 | 9.234 | -0.06 | 0.4 | 9.792 |
| -0.03 | 0.4 | 10.925 | -0.03 | 0.4 | 10.464 |
| 0 | 0.4 | 11.4 | 0 | 0.4 | 11.424 |
| 0.03 | 0.4 | 10.944 | 0.03 | 0.4 | 10.4832 |
| 0.06 | 0.4 | 10.108 | 0.06 | 0.4 | 9.5424 |
| 0.09 | 0.4 | 9.31 | 0.09 | 0.4 | 8.16 |
| 0.12 | 0.4 | 7.885 | 0.12 | 0.4 | 9.888 |
| 0.15 | 0.4 | 6.84 | 0.15 | 0.4 | 10.752 |
