# Supplementary material for: Large-scale experimental investigation of biotreated sand column using different grouting pipe configurations
Source: PLoS One. 2026 May 26;21(5):e0349797. doi: 10.1371/journal.pone.0349797 (PMC13210374; doi:10.1371/journal.pone.0349797)
Supplement: S6 Table — (DOCX) [file pone.0349797.s006.docx]

**S6 Table. Raw data corresponding to Fig 10**

| Figure 10(a) | | |
| --- | --- | --- |
| Layer | Penetration depth (mm) | Deviation (mm) |
| 1 | 8.815 | 2.25576 |
| 2 | 4.603 | 1.24184 |
| 3 | 5.03308 | 1.1391 |
| 4 | 4.78273 | 2.58086 |
| 5 | 10.25769 | 3.41778 |
| 6 | 9.98333 | 2.19899 |
| 7 | 14.29182 | 3.86714 |
| 8 | 15.72667 | 1.6958 |
| 9 | 16.636 | 0.90307 |
| 10 | 13.85444 | 2.65413 |
| 11 | 14.358 | 2.90277 |
| 12 | 12.49143 | 1.88005 |
| 13 | 16.42286 | 2.05172 |
| 14 | 15.035 | 1.735 |
| Figure 10(b) | | |
| Layer | Penetration depth (mm) | Deviation (mm) |
| 1 | 6.97308 | 1.74045 |
| 2 | 6.29 | 0.60334 |
| 3 | 6.757 | 3.3028 |
| 4 | 7.94286 | 1.92928 |
| 5 | 9.345 | 3.08913 |
| 6 | 15.08364 | 1.91665 |
| 7 | 15.65667 | 2.14789 |
| 8 | 16.11333 | 2.90554 |
| 9 | 16.35 | 1.06 |
| 10 | 17.015 | 1.90698 |
| 11 | 16.505 | 3.06247 |
| 12 | 15.981 | 1.96631 |
| 13 | 16.18636 | 2.9724 |
| 14 | 14.90286 | 2.1802 |
