# Supplementary material for: Large-scale experimental investigation of biotreated sand column using different grouting pipe configurations
Source: PLoS One. 2026 May 26;21(5):e0349797. doi: 10.1371/journal.pone.0349797 (PMC13210374; doi:10.1371/journal.pone.0349797)
Supplement: S7 Table — (DOCX) [file pone.0349797.s007.docx]

**S7 Table. Raw data corresponding to Fig 11**

| Width (m) | Depth  (m) | Calcium Carbonate content  (%) | Width (m) | Depth  (m) | Calcium Carbonate content  (%) |
| --- | --- | --- | --- | --- | --- |
| 0.015 | 0.1 | 11.12867 | 0.015 | 0.8 | 12.7954 |
| 0.045 | 0.1 | 8.86848 | 0.045 | 0.8 | 7.86355 |
| 0.075 | 0.1 | 7.92536 | 0.075 | 0.8 | 8.59446 |
| 0.105 | 0.1 | 6.30123 | 0.105 | 0.8 | 10.32266 |
| 0.135 | 0.1 | 7.15955 | 0.135 | 0.8 | 7.67004 |
| 0.165 | 0.1 | 7.79584 | 0.165 | 0.8 | 4.83756 |
| 0.195 | 0.1 | 8.05577 | 0.195 | 0.8 | 5.839 |
| 0.225 | 0.1 | 7.78281 | 0.225 | 0.8 | 6.33373 |
| 0.255 | 0.1 | 9.75849 | 0.255 | 0.8 | 9.69963 |
| 0.285 | 0.1 | 11.30317 | 0.285 | 0.8 | 7.70088 |
| 0.015 | 0.2 | 11.8075 | 0.015 | 0.9 | 12.64473 |
| 0.045 | 0.2 | 9.91329 | 0.045 | 0.9 | 10.40037 |
| 0.075 | 0.2 | 8.80324 | 0.075 | 0.9 | 7.01398 |
| 0.105 | 0.2 | 9.09662 | 0.105 | 0.9 | 6.58932 |
| 0.135 | 0.2 | 11.29873 | 0.135 | 0.9 | 6.61008 |
| 0.165 | 0.2 | 11.75653 | 0.165 | 0.9 | 4.95946 |
| 0.195 | 0.2 | 9.64827 | 0.195 | 0.9 | 6.22461 |
| 0.225 | 0.2 | 7.99938 | 0.225 | 0.9 | 8.24114 |
| 0.255 | 0.2 | 9.32022 | 0.255 | 0.9 | 9.31452 |
| 0.285 | 0.2 | 12.1662 | 0.285 | 0.9 | 7.35708 |
| 0.015 | 0.3 | 13.10089 | 0.015 | 1 | 11.0175 |
| 0.045 | 0.3 | 10.7438 | 0.045 | 1 | 8.51328 |
| 0.075 | 0.3 | 10.5602 | 0.075 | 1 | 6.31976 |
| 0.105 | 0.3 | 9.48489 | 0.105 | 1 | 6.09281 |
| 0.135 | 0.3 | 6.82616 | 0.135 | 1 | 6.41904 |
| 0.165 | 0.3 | 13.4659 | 0.165 | 1 | 5.83549 |
| 0.195 | 0.3 | 10.49486 | 0.195 | 1 | 5.69258 |
| 0.225 | 0.3 | 10.68819 | 0.225 | 1 | 5.57398 |
| 0.255 | 0.3 | 13.40527 | 0.255 | 1 | 7.63271 |
| 0.285 | 0.3 | 14.7948 | 0.285 | 1 | 7.78661 |
| 0.015 | 0.4 | 14.4491 | 0.015 | 1.1 | 7.59942 |
| 0.045 | 0.4 | 9.94466 | 0.045 | 1.1 | 8.62932 |
| 0.075 | 0.4 | 8.50513 | 0.075 | 1.1 | 5.15908 |
| 0.105 | 0.4 | 7.43373 | 0.105 | 1.1 | 4.16305 |
| 0.135 | 0.4 | 8.23658 | 0.135 | 1.1 | 3.71026 |
| 0.165 | 0.4 | 8.10615 | 0.165 | 1.1 | 3.4794 |
| 0.195 | 0.4 | 9.10244 | 0.195 | 1.1 | 2.76319 |
| 0.225 | 0.4 | 11.60378 | 0.225 | 1.1 | 3.35989 |
| 0.255 | 0.4 | 13.55586 | 0.255 | 1.1 | 8.32046 |
| 0.285 | 0.4 | 14.59633 | 0.285 | 1.1 | 7.90111 |
| 0.015 | 0.5 | 13.09786 | 0.015 | 1.2 | 7.19686 |
| 0.045 | 0.5 | 8.60505 | 0.045 | 1.2 | 6.95672 |
| 0.075 | 0.5 | 7.75513 | 0.075 | 1.2 | 3.08605 |
| 0.105 | 0.5 | 6.54726 | 0.105 | 1.2 | 3.8524 |
| 0.135 | 0.5 | 7.897 | 0.135 | 1.2 | 4.62425 |
| 0.165 | 0.5 | 8.56562 | 0.165 | 1.2 | 4.6857 |
| 0.195 | 0.5 | 7.74798 | 0.195 | 1.2 | 4.79592 |
| 0.225 | 0.5 | 10.48621 | 0.225 | 1.2 | 5.56934 |
| 0.255 | 0.5 | 14.57029 | 0.255 | 1.2 | 7.533 |
| 0.285 | 0.5 | 10.24673 | 0.285 | 1.2 | 9.27311 |
| 0.015 | 0.6 | 12.12092 | 0.015 | 1.3 | 3.0803 |
| 0.045 | 0.6 | 14.66679 | 0.045 | 1.3 | 2.39389 |
| 0.075 | 0.6 | 11.49408 | 0.075 | 1.3 | 3.22592 |
| 0.105 | 0.6 | 7.37492 | 0.105 | 1.3 | 4.03451 |
| 0.135 | 0.6 | 7.72604 | 0.135 | 1.3 | 4.4148 |
| 0.165 | 0.6 | 6.13892 | 0.165 | 1.3 | 3.21386 |
| 0.195 | 0.6 | 8.70057 | 0.195 | 1.3 | 3.10917 |
| 0.225 | 0.6 | 10.21027 | 0.225 | 1.3 | 3.36325 |
| 0.255 | 0.6 | 10.99113 | 0.255 | 1.3 | 3.67684 |
| 0.285 | 0.6 | 12.67171 | 0.285 | 1.3 | 4.37485 |
| 0.015 | 0.7 | 17.05986 | 0.015 | 1.4 | 3.80429 |
| 0.045 | 0.7 | 9.38429 | 0.045 | 1.4 | 4.4909 |
| 0.075 | 0.7 | 6.79681 | 0.075 | 1.4 | 5.22783 |
| 0.105 | 0.7 | 6.73287 | 0.105 | 1.4 | 5.61326 |
| 0.135 | 0.7 | 8.11149 | 0.135 | 1.4 | 3.64791 |
| 0.165 | 0.7 | 6.18 | 0.165 | 1.4 | 2.95691 |
| 0.195 | 0.7 | 7.75114 | 0.195 | 1.4 | 2.88432 |
| 0.225 | 0.7 | 9.49964 | 0.225 | 1.4 | 3.42111 |
| 0.255 | 0.7 | 12.59603 | 0.255 | 1.4 | 4.2423 |
| 0.285 | 0.7 | 12.11897 | 0.285 | 1.4 | 4.90508 |
